# Assessment and Monitoring of Fish Quality from a Coastal Ecosystem under High Anthropic Pressure: A Case Study in Southern Italy

**DOI:** 10.3390/ijerph17093285

**Published:** 2020-05-08

**Authors:** Giovanna Loredana La Torre, Nicola Cicero, Giovanni Bartolomeo, Rossana Rando, Rossella Vadalà, Antonello Santini, Alessandra Durazzo, Massimo Lucarini, Giacomo Dugo, Andrea Salvo

**Affiliations:** 1 Department of Biomedical and Dental Sciences and Morphofunctional Imaging, University of Messina, Polo Universitario Annunziata, 98168 Messina, Italy; llatorre@unime.it (G.L.L.T.); gbartolomeo@unime.it (G.B.); rrando@unime.it (R.R.); rosvadala@tiscali.it (R.V.); dugog@unime.it (G.D.); 2 Science4Life, Spin Off Company, University of Messina, V.le Leonardo Sciascia Coop Fede Pal. B, 98168 Messina, Italy; 3 Department of Pharmacy, University of Napoli Federico II, Via D. Montesano 49, 80131 Napoli, Italy; asantini@unina.it; 4 CREA-Research Centre for Food and Nutrition, 00178 Roma, Italy; alessandra.durazzo@crea.gov.it (A.D.); massimo.lucarini@crea.gov.it (M.L.); 5 Dipartimento di Chimica e Tecnologie del Farmaco, Università La Sapienza, P.le Aldo Moro 5, 00185 Roma, Italy; andrea.salvo@uniroma1.it

**Keywords:** fish, heavy metals, food safety, human health, pollution, Mediterranean Sea

## Abstract

Fish is a nutrient-rich food but, at the same time, consumption of fish is a possible source of exposure to heavy metals. Since many coastal Mediterranean areas suffer from great anthropomorphic pressure, the aim of this study was to assess the level of potentially toxic inorganic elements in different fish samples from the coastal zone of Southern Italy (Gela) where there is a high mortality rate linked to cancer disease and congenital malformations. The presence of mercury, cadmium, lead, nickel, arsenic, vanadium, and chromium was measured by inductively coupled plasma-mass spectrometry (ICP-MS). The risk assessment was evaluated in terms of estimated daily intake by calculating the amount of potentially toxic elements that an average individual adult weighing 60 kg would ingest. Moreover the non-carcinogenic risk was estimated by target hazard quotient (THQ). The study evidenced significant contamination by inorganic elements, especially cadmium, which can be linked to industrial pollution. The THQ indexes, as indicators of human health, suggest that the consumption of fish from the study area is not free of risk.

## 1. Introduction

Pollution due to potentially toxic inorganic elements affects many coastal regions of the world [1,2,3]. These chemicals are, in fact, not biodegradable and accumulate up to the trophic levels of the food chain, thus producing adverse effects on the marine ecosystem [4]. On the South coast of Sicily, there is the most striking example of a compromised landscape in regards to the ecological network, as the Gela petrochemical industry is the main cause of marked chemical contamination in the South-East of Sicily [5], which should be one of the most attractive areas of the Region. Yet, since 1960 the industrial development has generated such serious environmental and health-related damage that in 1990, that the Gela municipality and two neighboring municipalities (Niscemi and Butera) were included among the areas at high risk of environmental crisis and, in 1998 an extensive area of Gela municipality was designated as a Gela Reclamation Site of National Interest (RSNI) for soil remediation [6,7,8,9,10].

The activities of the industrial district of Gela, an area of Italy where the quantity and danger of pollutants requires remediation, have led to a progressive contamination of the environment with extremely high levels of toxic, persistent and bioaccumulating pollutants [11,12]. For some companies that have carried out the maintenance work of the pipelines for the embarkation and disembarkation of petroleum products, the sea has become an immense illegal landfill to be used for significant savings in the costs of special waste disposal. Thus, tons of scrap iron, metal drums and building material waste are stacked on the seabed near the Gela refinery, along the pipe which, with the breakwater, makes up the former petrochemical port. Eliminating this waste material would take several years of work, and imply considerable economic investments [13].

Many important projects have been proposed to reduce the risk and dramatic healthcare consequences caused by the Gela refinery over more than half a century. In fact, following the Marine Strategy Framework Directive [14], which establishes a framework for marine environmental policy that aims to achieve good environmental status (GES) of the EU’s marine waters by 2020 [15,16,17], the Sicilian Region Authority for the Landscape and Environment started redrafting the management plan for this site [18]. This requires improving current assessment capabilities, through the characterization of the marine-coastal environment by suitable indicators of contamination.

Since 1998, preliminary studies conducted by the interdisciplinary group of oceanography of the National Council of Research focused on the state of health of the central basin of the Mediterranean Sea, the area of the Strait of Sicily and the area of Gela. Most of these studies concerned the mineral distribution in both the dissolved phase and the suspended particulate of seawater samples collected at different depths, characteristics of the foraminifera found and inorganic pollutants in sediments [19,20,21,22,23]. These investigations help in determining the magnitude of anthropogenic contamination and particular attention has been paid to pollutants migration in the environment and to the potential exposure of the population; however, they do not provide any information about the biota’s contamination. It is well known that inorganic contaminants, such as heavy metals, are of particular concern due to their environmental persistence and potential ecological risks. Moreover, heavy metals can interact with many aquatic organisms that can assimilate dissolved metals directly, causing unwanted bioaccumulation.

Recently, our research group evaluated the pollution level induced by potentially toxic inorganic elements in vegetables coming from the area of Gela and the results suggested that the concentrations of some elements (particularly cadmium and arsenic) might determine important cancer risk for the consumers [10]. Considering that toxic inorganic pollution in the Gela area should not be neglected and, despite the fact that in 2014 the petrochemical plant was reconverted into a “green” refinery, Gela citizens risk dramatic health consequences generated by the activity of the refinery during the last fifty years. This study aims at monitoring the accumulation of potentially toxic inorganic pollutants in different fish samples collected in the Gulf of Gela. The simultaneous determination of some inorganic elements (Hg, Pb, Cd, Cr, Ni, Cu, V, and As) by inductively coupled plasma mass spectrometry (ICP-MS) in fishes and sediments represents a global tool to assess the marine environmental safety of Mediterranean Sea close to Gela coasts and, at the same time, can be considered from a nutritional standpoint to evaluate the risk associated with seafood consumption for human health.

## 2. Materials and Methods

### 2.1. Study Area

**Figure 1:**
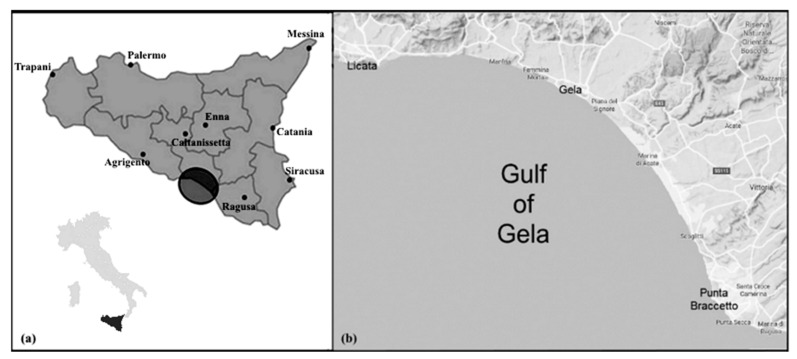
Location of (**a**) Gela area in Sicily (Italy) and (**b**) study area of Gela and municipalities of the high environmental risk area.

The Gulf of Gela is located on the Southern coast of Sicily and is the largest gulf of the island. It extends for about 25 km and is included between Punta Braccetto to the East, and Licata to the West (Figure 1). The coast, characterized by typical dunes with Mediterranean vegetation, is low and sandy. In short stretches, the sandy shore shows high ecological diversity in terms of environmental heterogeneity and variability of species composition. The whole coast overlooks the gulf, has no natural ports and the seabed is generally low. Nevertheless, in the gulf there are some artificial ports: the port “Rifugio di Gela” (used for small boats), the port “Isola di Gela” (used by the petrochemical refinery, especially for tankers), the port of Licata and the port of Scoglitti.

The entire coastal stretch has a low soil attenuation capacity, i.e., that of filtering against potential pollutants [12]. The area presents an intrinsic vulnerability of the aquifer to pollution. In fact, due to the high permeability of the wind sands and the presence of groundwater, the pollutant, if present, reaches the surface in a short time, which is not sufficient for a suitable self-purification. Therefore, the geological study of the coastal stretch, following an approach based on pollution, attributes a medium-high degree of vulnerability to this area [24].

Pollution in the study area dates back to industrial activities that have been carried out since the mid-1950s, which exposed the soil and the aquifer to contamination from anthropogenic origin [10]. The main causes of pollution of the coastal marine environment in the Gulf of Gela are linked to the discharge of process and cooling waters of the industrial pole, the port activities, the delivery of poorly or not purified municipal sewage to the sea, and the delivery of waste water from agricultural land to the sea [8]. Several rivers and streams flow into the Gulf of Gela and some of these are near the refinery. This one is located in a flat area in the middle of the Gulf of Gela and, more precisely, in the east of the river Gela whose flow rate is strongly influenced by the discharge of cooling water from the petrochemical plant. Moreover, about 5.5 km from the refinery in the East, there is the Acate river, whose environmental status has been defined as “bad” since the water quality standard—expressed as the maximum permissible concentration for some chemical pollutants in the water—exceeds the quantitative level of even a single pollutant. [25].

The seabed in front of the refinery consists of medium and coarse sands (between 0.18 and 2 mm), and is characterized by a reduced slope of about 1.5% and up to 2%. The coasts of the study area have become more vulnerable to natural hazard related to intense erosion caused by the sea. The coastline is exposed to wave action, especially from North-West to South-East; also, the marine currents within the Algerian current of the Strait of Sicily are characterized by prevalent NW–SE direction.

### 2.2. Reagents and Standards

The gases, 99.9990% argon and 99.9995% helium, were supplied by Rivoira gases (Rivoira S.p.A., Milan, Italy). Concentrated (65%, v/v) nitric acid trace metal analysis grade (J.T. Baker, Mallinckrodt Baker, Milan, Italy) and concentrated (30%, v/v) hydrogen peroxide (J.T. Baker, Mallinckrodt Baker, Milan, Italy) were used to digest samples. Ultrapure de-ionized water (resistivity l8.2 MΩ. cm) was obtained from a Milli-Q water purification system (Thermo Scientific Barnstead Smart2Pure 12—Milan, Italy). Stock standard solutions (1000 mg L^−1^ in 2% nitric acid) of each element under investigation were purchased from Fluka, Milan, Italy (Cr, Ni, Cu, Pb, As, and V) and from Merck, Darmstadt, Germany (Hg and Cd). Also, standard solution of Re at 1000 mg L^−1^ in 2% nitric acid was acquired by Fluka (Milan, Italy) and used, as preparation standard at 0.8 mg L^−1^, to verify the sample digestion and to correct the volumetric changes. Stock standard solutions of ^115^In, ^45^Sc, ^103^Rh, and ^209^Bi (1000 mg L^−1^ in 2% nitric acid) were purchased from Fluka (Fluka, Milan, Italy) and were used as on-line internal standards (at level of 10 μg L^−1^) to correct instrumental drift and variations due to the matrix. To tune the instrument, an ICP-MS tuning solution containing ^59^Co, ^7^Li, ^80^Y and ^205^Tl (each 1.0 μg L^−1^ in 2% HNO_3_) was obtained from Agilent (Santa Clara, CA, USA). Mixed working calibration standards were prepared at concentration ranges suitable for the analytes being investigated. Before use, all glassware was washed with 5% HNO_3_ for at least 12 h, rinsed with ultrapure water and then dried.

### 2.3. Sampling

Ten fish samples and ten sea urchins were collected in the Gulf of Gela in order to carry out a bio-monitoring of the marine environment. The fish samples were netted and obtained from local fishermen between the east area of the petrochemical site and the city of Gela, while the sea urchins were taken manually from the same place. The sampling took place between March–April 2018. The physicochemical properties, e.g., pH (8.35 at 18.5 °C) and dissolved oxygen (187.60 mg L^−1^) of seawater were determined. In detail, the samples were as follows: 6 red mullets (*Mullus barbutus*) which were divided into two groups according to the their size and weight, namely: Group Mullett 1, with lengths 14–16 cm and weights 53–61 g, and group Mullett 2, with lengths 18–20 cm and weights 87–97 g; three sea hen (*Chelidonichthys lucernus*) with lengths between 25 and 30 cm and weights between 250–280 g; 1 ray (*Raja species*), 36 cm long and weighing 349 g; 10 sea urchins (*Paracentrotus lividus*) with diameters between 55 and 60 mm collected at a depth between 2 and 7m.

The analytical data of the fish samples and sea urchins from Gela were compared with other fish samples caught in Sicilian areas where there is no industrial settlement. These samples were used as controls to assess the significance of the different levels of contaminants observed.

For fish samples, the comparison was performed on 8 fish specimens: 2 specimens of sand streenbras (*Lithognathus mormyrus*) caught by net in Marina di Ragusa, an area with seabed and marine environments similar to those of the Gulf of Gela and where there are no industrial settlements, 3 sea bass (*Dicentrarchus labrax*) and 3 sea bream (*Sparus aurata*) taken from a breeding facility, located in the Gulf of Patti in the Tyrrhenian Sea (North of Sicily) and unaffected by human activities. Each fish sample was subjected to biometric measurements (weight, total length, height, diameter for the sea urchins) and stored in a plastic food bag at −80°C until dissection and analysis.

In order to carry out a bio-monitoring of the marine environment, since the sea urchin (*Paracentrotus lividus*) is a suitable organism to fill the role of indicator due its wide distribution, easy collection, filter-feeding habits and good tolerance to pollutants [26,27,28,29,30,31], 20 sea urchins (*Paracentrotus lividus*) were manually collected from two control areas: Marina di Ragusa (10 specimens) and Piraino in the province of Messina (10 specimens). The sea urchin gonads were extracted, homogenized, placed in test tubes and transferred to the freezer at −20 °C until analysis.

**Figure 2:**
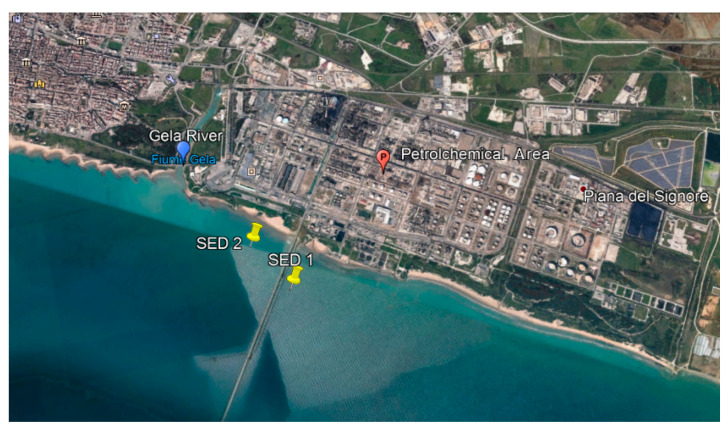
Sampling plan of marine sediments in the Gulf of Gela.

In order to obtain any indicative data on the contamination of marine sediments in the area in front of the Gela refinery, in the same period, 2 samples of surface sediments were collected, for a total weight of about 1 kg for each sample (Figure 2).

Each sample was formed by the union of 5 superficial samplings (0–10 cm depth), performed randomly at a depth of 3 to 6 m, each within a square mesh of 50 m × 50 m. The sediment sample SED 1 was taken about 1 km from the shore (about 4 m deep) while the sediment sample SED 2 was taken about 500 m from the shore (about 3 m deep).

The samples were homogenized *in situ* and stored, separately, inside polyethylene containers (Sarstedt, Germany). After collection, the samples were stored at 0 °C. Coordinates, depth of the seabed, color, odor and type of sediment were recorded (Table 1).

To minimize sample contamination, all sample handling was performed wearing disposable, powder-free, latex gloves in clean laboratory areas.

### 2.4. Sample Pre-Treatment

#### 2.4.1. Sediment Samples

Initially, the sediment samples were dried in an oven at 40 °C till constant weight; then a part of each sample was powdered using an agate mortar and an agate pestle. About 1 g of dry powdered sample was digested with 2.5 mL of HNO_3_, 0.8 mL of HCl, 1 mL H_2_O_2_, and 1mL of Re internal standard in Teflon containers. The mineralization was carried out using a microwave oven (Mileston ETHOS 1, Milestone, CT, USA) with a constant microwave power (1000 W). The heating program was as follows: temperature was increased to 200 °C in 10 min (Step1), and then it was held to 200 °C for 10 min (Step 2). After the mineralization, the residues were cooled at room temperature, filtered and diluted to 25 mL with ultrapure water. All the final samples were analyzed by ICP-MS.

#### 2.4.2. Fish Samples

Each fish sample was dissected and the muscle tissues, without skin, were collected and homogenized with a ceramic knife to constitute the global sample. About 0.7 g of the homogenized sample were placed in a Teflon vessel and added with 7 mL of 65% HNO_3_, 1 mL of 30% H_2_O_2_, and 1mL of Re internal standard. The samples were digested using an optimized method (Step 1 for 10 min at 200 °C and Step 2 for 20 min at 200 °C with a power of 1000 watts), and then made to volume in 25 mL ultrapure water. To minimize the error in the mineralization phase, a blank sample was prepared and subjected to the same procedures.

### 2.5. ICP-MS Analysis

The determination of the minerals was carried out using the same procedure adopted for the determination of potentially toxic inorganic species in vegetables and zebra fishes [10,32]. The digested samples were analyzed using an Agilent 7500CX ICP-MS spectrometer (Agilent, Santa Clara, CA, USA) with Octapole Reaction System (ORS), reaction/collision cell, and an ASX 500 auto sampler. The system was pressurized with helium to remove the interference due to the plasma and to the matrix. The analyses were performed either in gas mode or in no gas mode.

The ICP-MS operating conditions were the following: RF power, 1550 W; plasma gas f1ow rate, 15 L min^−1^; auxiliary gas flow rate, 0.9 L min^−1^; carrier gas flow rate, 1.1 L min^−1^; sample introduction flow rate, 1 mL min^−1^; sample depth, 9 mm; spray chamber temperature, 2 °C; vacuum, <1.5 × 10^−7^ Pa; interface pressure; 5.3 × 10^−2^ Pa. These parameters were optimized using a solution containing nuclides ^7^Li, ^59^Co, ^80^Y and ^205^Tl (10 μg L^−1^), whose masses were distributed all over the interval of interest.

For each element, selected isotopes were chosen on the basis of the relative isotopic abundance, in order to optimize the sensitivity, and the absence of important isobaric interferences or interference induced by the matrix, when possible. They were the following: ^63^Cu, ^202^Hg, ^114^Cd, ^60^Ni, ^75^As, ^51^V, ^52^Cr and ^208,207,206^Pb.

A solution of ^115^In, ^45^Sc, ^103^Rh and ^209^Bi (10 µg L^−1^ final concentration) was used as an on-line internal standard to correct any instrumental drifts and matrix effects.

Quantitative measurements were carried out using the external standard method. The calibration was performed with a multi-standard solution of Cr, V, Cu, Cd, Pb, Ni and As obtained by mixing standard solutions of each individual element and diluting with nitric acid (HNO_3_, 2%). Six standard mixtures of all the elements, at different concentrations ranging from 5 to 2000 μg L^−1^ for each element, were prepared by adding the appropriate amount of the element standard solution in 10 mL volumetric flasks and bringing to volume with HNO_3_ 2%.

In all the samples Hg was analyzed separately, applying a procedure that we used in an earlier study [10]. Therefore, to minimize any memory effects, a washing solution with HNO_3_ 2%, was fluxed between an analysis and the subsequent.

In order to exclude error and to satisfy quality assurance, all analyses were carried out in triplicate, including blank and certified reference material.

### 2.6. Quality Assurance

The analytical process used was validated according to the regulation [33] and international guidelines [34,35]. The evaluation of the linearity was based on injection of five standard solutions. Each solution was injected six times (n = 6). Good linearity was observed in each concentration range, with R^2^ always >0.99993. The limits of detection (LODs) and of quantification (LOQs) were calculated according to the International Union of Pure and Applied Chemistry (IUPAC) guidelines [36] and Table 2 reports the validation parameters. 

The limits of detection (LODs) and of quantification (LOQs) were experimentally calculated as 3.3 σ/S and 10 σ/S, respectively, where σ is the residual standard deviation and S is the slope of the calibration curve. LOD values ranged from 0.0004 to 0.006 μg L^−1^, while LOQ values ranged from 0.001 to 0.02 μg L^−1^.

Accuracy was assessed by evaluating six determinations on certified reference materials and was reported as recovery (%) between the value found with the calibration curve and the true value reported, together with the relative standard deviation percentage (RSD%). The accuracy for the sediments was evaluated on the certified matrix SOIL, GBW07402 provided by Natural Resources Canada (NRCan) certificate for As 13.7 mg kg^−1^, Cd 0.071 mg kg^−1^, Cr 47 mg kg^−1^, Cu 16.3 mg kg^−1^, Pb 20 mg kg^−1^, Ni 19.4 mg kg^−1^, V 62 mg kg^−1^, Hg 15 ng g^−1^. Natural Resources Canada (NRCan) helps improve the reliability of measurements at mineral analysis labs in Canada and around the world. The certified matrix used for fishes was FISH TISSUE IAEA—407 provided by IAEA Environment Laboratories and certified for As 12.6 mg kg^−1^, Cd 0.189 mg kg^−1^, Cr 0.73 mg kg^−1^, Cu 3.28 mg kg^−1^, Pb 0.12 mg kg^−1^, Ni 0.60 mg kg^−1^, V 1.43 mg kg^−1^, Hg 0.222 mg g^−1^. The IAEA Environment Laboratories produce certified reference materials for the measurement of radionuclides, trace elements and organic contaminants. The obtained results, reported in Table 3, show that the recovery on the certified matrix GBW07402 varies from 87 to 112% with Relative Standard Deviation (RSD) values always lower than 5.62%. For the matrix FISH TISSUE IAEA—407, the recovery varied from 75 to 101% with RSD values always lower than 7.23%.

The precision was also evaluated by intra-day and inter-day repeatability. The intra-day repeatability of the method was assessed from replicates of the same material analyzed in the same batch (*n = 10*) and in different days (*n = 20*). The precision, theresults of which are reported as RSD% in Table 3, was excellent in the same analytical run (RSD% between 0.8 and 3.7 for fish and between 2.3 and 3.8 for soil) and good for long-term analysis (RSD% between 2.7 and 7.1 for fish and between 3.0 and 9.5 for soil).

### 2.7. Target Hazard Quotient Calculation

The non-carcinogenic effect, expressed as target hazard quotient (THQ) and defined as the ratio of the potential exposure to a substance and the level at which no adverse effects are expected, was determined following the Equation (1):

THQ = 10^−3^ (Efr × ED_tot_× FIR × C) / (RfD_o_× BWa × ATn) (1)

where Efr is the exposure frequency (365 day/year), ED_tot_ is the exposure duration (70 years), FIR is the average daily consumption of muscle meat of fish or sea urchins (gr/day), C is the average concentration of the heavy metals in the sample (mg/kg), RfDo is the oral reference dose (mg/kg-day), BWa is the average body weight (kg) and ATn is the average exposure for non-carcinogens in a year (365days/year of exposure, assuming 70 years in this study). The data were estimated for an Italian adult (body weight 60 kg) that ingests 77.8 gr of fish [37] and 4 gr of sea urchins daily. Particularly, since daily ingestion values are not reported for sea urchins, and for the reason that European people generally do not eat them in the same amount as fishes but as mollusks, we assumed a weekly ingestion of sea urchins corresponding to 30 gr (about 4 gr per day).

A THQ value below 1 indicates no adverse effect for human health; if THQ is greater than 1, then adverse health effects are possible.

Moreover, considering that exposure to more pollutants may cause cumulative and/or interactive risk effects, based on United States Environmental Protection Agency (USEPA) suggestions [38], we calculate the Combined Target Hazard Quotient (CTHQ) according to the Equation (2):

(2) $$\mathrm{CTHQ}\;=\overset{n}{\underset{k=1}{\sum }}\mathrm{THQ}$$

where *n* = 1, 2, …, n is the individual THQ for the studied inorganic elements.

## 3. Results and Discussion

### 3.1. Level of Potentially Toxic Inorganic Elements in Marine Sediments

The concentrations of the inorganic elements (As, Cd, Cr, Cu, Hg, Ni, Pb and V) in sediments from the investigated area are shown in Table 4. The study, involving only two sediments, revealed that both have high concentrations in V (14.89 mg kg^−1^ and 27.17 mg kg^−1^), As (10.76 mg kg^−1^ and 13.44 mg kg^−1^) and Ni (5.37 mg kg^−1^ and 11.11 mg kg^−1^) of apparent industrial origin.

The highest concentrations of potentially toxic elements in the sediments have been found in the internal area of the Port due to a lower dispersion capacity of the sedimented material and a greater contribution of municipal sewage containing such pollutants. In fact, as data shows, the levels of potentially toxic inorganic elements had a different distribution profile in the two samples tested and the sediment SED 2, characterized by a dark-gray color and the persistent odor of hydrocarbons and hydrogen sulfide, showed higher concentrations of potentially toxic elements. The analytical data indicate that the levels of Pb, Cr, Ni, V and Cu in SED 2 were approximately two times higher than those of SED 1 and these results evidence a progressive increase in concentration of potentially toxic species from the outermost zone to the innermost one. In addition, the levels of Pb and Ni in SED 1 were comparable with previous studies in the Strait of Sicily [39]. In both sampling sites, the Cd levels were significantly exceeded (about 10 times) with respect to the Coppola [39] investigation, indicating anthropogenic contamination of sediments in the area.

To evaluate the pollution and assess the sediment quality, the classification of the Environmental Quality Status (EQS) of sediments was held according to the indications of the European directive [40], implemented with DL 152/2006 and DM 260/2010 [41,42]. Comparing the results with the level established by the Ministerial Decrees [42] we highlight that Cd in sediments shows the most serious contamination among all the potentially toxic inorganic contaminants considered, exceeding about two time the limits in both samples. Contamination degree of As in sediment was also high, especially in the SED 2 sample in which the legal limits were exceeded. Nevertheless, these levels were lower than those reported for other strongly anthropized port areas of Sicily [43] and comparable with those recently observed in the Strait of Sicily [39]. Most likely, the discovery of potentially toxic elements in the sediments is possibly due to impacts deriving from specific anthropic stresses such as the presence of large industrial settlements that persist on the Strait of Sicily. However, the environmental outcome and the spread of potentially toxic elements in the sediment is linked to the performance of multiple competing variables such as: settling times of individual metals, effects of migration currents along the low sandy coast of the Strait of Sicily, sediment accumulation times linked to the sediment granulometry, preferential bioaccumulation processes for some metals (e.g., lead and cadmium) in living organisms. Probably, the presence of mercury in the sediments of the study area is attributable to the activities linked to the Chlorine-Soda plant in Gela, now closed, which for years poured mercurial residues into the sea. The low content in the analyzed sediments can be explained considering that the mercury possibly stratified in deeper layers than those collected. The high vanadium content is most likely not only due to its natural origin and could also be attributed to the petroleum activity of Gela. Generally, we could assume that in this marina area a correlation exists among the concentrations of potentially toxic elements that were present in sediments in higher levels. It may be inferred that such a relationship could be more significant in locations which are more affected by anthropogenic sources, in this case petroleum derivatives and antifouling paints used for boats in the Gulf of Gela.

### 3.2. Level of Potentially Toxic Inorganic Elements in Sea Urchins

The results on the considered potentially toxic elements in *P. Lividus gonads* collected from Gela are shown in Table 5. The data were compared with control samples from areas not contaminated by industrial activities such as “Marina di Ragusa” and “Piraino”. All the data regarding the concentrations of inorganic elements in fish samples are expressed in mg kg^−1^ of wet weight, as indicated by the Commission Regulation N°1881/2006 [44].

The 30 sea urchin samples were analyzed and grouped by geographic area, each group consisting of 10 samples. As the data show, the samples from Gela were the most contaminated and the average concentration values of potentially toxic elements in the sea urchins from this area were in the order: Pb> As> Cr> Ni> Hg≥ Cd. Moreover, the values evidenced that sea urchins from Gela had the highest mean concentrations of As (6.17 mg kg^−1^), Cu (2.31 mg kg^−1^) and Cr (2.29 mg kg^−1^), compared to samples from “Marina di Ragusa” and “Piraino”. On the contrary, the Cd content (0.02 mg kg^−1^) was about 60 times lower than that found in samples from “Marina di Ragusa” in which V (0.94 mg kg^−1^) and the highest mean levels of Ni (1.18 mg kg^−1^) were determined.

The scientific literature indicates that *P. Lividus* is an organism suitable to cover the role of biological-biochemical indicator due to its wide distribution, easy collection, alimentary and filtering habits, and good tolerance to polluting substances. It has been used in several local pollution studies as a bioindicator of heavy metal contamination in the marine environment [29,30,31,45,46,47,48].

Besides being compatible with the results of the survey carried out in this study on marine sediments, the inorganic contaminants detected in the sea urchin gonads and, in particular, the levels of Ni, V, As and Hg have a possible anthropogenic origin and could be linked to the activities of the Petrochemical plant. The samples taken at “Piraino” were least contaminated and this is consistent with the absence of industrial anthropogenic activities; instead, contrary to what we expected, the samples from “Marina di Ragusa” were not absolutely pristine and this could probably be due to the high vessel traffic, especially during the sampling.

The concentrations determined for Pb and Hg in urchins from Gela were higher as compared to some data in the literature [45,47,48], however, our values were comparable to those determined by Salvo et al. [29] in other urchin samples from Gela. The Cd levels in Gela samples were on average about 10 times lower than the values reported by Warnau et al. [48] but comparable with other data [47]. The Cr content, on average, was comparable to an earlier report [48] and to values determined by Salvo et al. [29]. Cu concentrations in urchins from Gela were much higher than those reported by Warnau et al. [48] but comparable with other data from the Mediterranean area [29,47]. The Ni and V results in *P. Lividus* from Gela were comparable with previous data determined by Salvo et al. [29] which, furthermore, are the only data reported in the literature for the Mediterranean area.

European food legislation does not set any limits for heavy metals in sea urchins; however, after comparing the content of Pb, Cd and Hg with the limits established by the European Community for bivalve mollusks [44,49], it emerged that Pb in sea urchin samples from Gela was 15 times higher than the limits established for fish safety criterion (1.5 mg kg^−1^). On the other hand, according to the regulation, the investigated samples from Gela were characterized by mean concentration of Hg and Cd (0.03 and 0.02 mg kg^−1^, respectively), well within the maximum content set at 0.5 and 1.0 mg kg^−1^ for mercury and cadmium, respectively.

### 3.3. Level of Potentially Toxic Inorganic Elements in Fishes 

The concentration of potentially toxic elements in fish samples allow us to provide double information: one of environmental nature linked to the behavior of some species as bio-indicators, and another one linked to the food safety of the product intended for human consumption. Therefore, to rationalize the results of the research on fish samples, the data were divided into two Tables.

In Table 6, the analytical results regarding the muscles of the collected mullets (*Mullus barbatus*) are grouped together. This species has a high economic and commercial interest for the Gela city. It is a benthic and territorial fish and is indicated by FAO/UNEP [50] as a species suitable for monitoring heavy metal pollution. The samples of mullets have been divided into two groups according to their size (Mullet 1 and Mullet 2) since the dimensions are recognized to be very important in the processes of absorption, distribution and elimination of pollutants [51]. The comparative analysis of potentially toxic elements between the two mullet groups confirms the importance of the dimensions in the metals accumulation: larger specimens (Mullet 2) show higher concentrations than the smallest ones (Mullet 1). This confirms that bioaccumulation is higher as the size increases, or in other words, old and larger fish generally have higher pollutant levels than young and small specimens [3,52,53].

The average concentration values (Table 6) of the inorganic elements detected in the mullets were in the following order: As > Cd > Pb > Ni > Cr > V > Hg > Cu.

The mean concentration of inorganic elements detected in *Mullus b.* from Gela were compared with the data obtained in previous studies carried out on the same species, with similar dimensions and in uncontaminated areas of the Mediterranean Sea [53,54,55,56], as well as with the law limits available, currently only for Cd, Hg and Pb [44,49,57].

The Cd concentrations in mullets from Gela were much higher than those reported in other works [53,56], indicating a possible effect of anthropogenic input on fishes. Thus, cadmium released from human activities such as fuel combustion, application of phosphate fertilizers or sewage sludge could accumulate in fishes. Because it is a non-degradable and cumulative pollutant, cadmium generally accumulates mainly in fish entrails (intestines, liver and kidneys), and from here it spreads later to fish muscles [58]. Concerning the toxicological value, the average Cd level (0.43 mg kg^−1^) in the samples from Gela was about 8.6 times higher than the legal limits established by the Commission Regulation N.488/2014 [49], amending the Regulation N. 1881/2006 [44] and fixing the limits for muscle meat of fish at 0.05 mg kg^−1^.

The Pb levels (average value 0.36 mg kg^−1^) were twice as high in the Gela samples compared to the data in the literature that refers to uncontaminated areas; however, this value does not differ much from the legal limit (0.30 mg kg^−1^) [44]. Among the potentially toxic elements that negatively impact the health status of marine environment, Hg deserve particular attention because its concentration (average value 0.07 mg kg^−1^) remains lower than the samples from Capo Passero [53] and below the legal limits fixed for muscle meat of fish (1.0 mg kg^−1^) [57].

Among the other micro-constituents for which no specific law limits exist, As deserves particular consideration as the concentration of this element in mullets was higher (average value 8.91 mg kg^−1^) than the other potentially toxic elements; moreover, the value was not comparable with the literature because this element has never been considered before. However, the current As levels suggest that the high concentrations of As, found also in marine sediments of the Gela area (12.09 mg kg^−1^), are probably of industrial origin and could influence the final As concentration in mullet.

Furthermore, Cr deserves more attention because, it is an essential element for the human body as it could be involved in the blood glucose metabolism [59,60]; however, when hexavalent and at high concentrations, chromium enters the bloodstream and can produce toxic, mutagenic and carcinogenic effects in biological systems [61,62]. Its presence in the environment is mainly linked to anthropogenic sources [53]. The comparative analysis of Cr concentrations shows that the levels are fairly homogeneous. Particularly, the results of the Gela samples are in agreement with those obtained by other authors in the Sicilian areas of Catania and Capo Passero [53].

As Table 6 shows, to the best of our knowledge, the V, Ni and Cu contaminations that we determined for mullets are not comparable with the data in the literature regarding the Mediterranean Sea. However, their levels in mullet muscles indicate that the possibility of inorganic element enrichment could arise in an area where there have been anthropogenic activities for many years. Particularly, as suggested by Antoniadis et al. [63], multi-element contamination cases are complex, and the enrichment of many elements is often highly related with the occurrence of other elements with similar chemical behavior. Therefore, multi-element contaminations cause more severe effects cumulatively than the enrichment of any given element alone.

Finally, Table 7 shows the results of the study carried out on fillets samples of other fish species caught in the Gulf of Gela: 2 sea hen (*Chelidonichthys lucernus*) and 1 ray (*Raja species*). The samples of Gela were compared with fishes of other species coming from areas (Marina di Ragusa and Patti) not subject to industrial contamination: 2 streenbras (*Lithognathus mormyrus*), 3 sea bass (*Dicentrarchus labrax*) and 3 sea bream (*Sparus aurata*). Considering the variability of potentially toxic elements accumulation among different fish species and in order to make the data comparable, the objective of the study was to examine the same species both in the survey area and in the control area, unfortunately this was not possible as for urchins (*Paracentrotus lividus*) or mullets (*Mullus barbutus*) for which we considered the data available in the literature. In spite of these difficulties, all of them are fishes that live and feed on or near the bottom of seas; moreover, the fish samples had similar lengths.

The survey on these last fish samples showed that Hg levels (range: 0.02–0.08 mg kg^−1^) were always below the limits established by the regulation (1.0 mg kg^−1^) [44,57] while Cd concentrations (range: 0.37–0.40 mg kg^−1^) were consistently far higher than the legal limits (0.050 mg kg^−1^) [49]. In particular, the two species caught in Gela (sea hen and ray) showed Cd levels up to eight times higher (0.39 and 0.40 mg kg^−1^, respectively), while the Pb contents (0.31 and 0.33 mg kg^−1^, respectively) were slightly above the legal limits (0.30 mg kg^−1^) [44]. What is particularly surprising is that these values were not very different from those determined for the specimens fished in Patti and Ragusa, areas which are far from direct anthropogenic impacts and are believed to be unpolluted and pristine. This result could be explained by the fact that in the Gulf of Patti there is a nature reserve (code ITA030012) in which a colony of yellow-legged gulls reside. Thus, in this area, a bird mediated contamination of trace elements (especially for As, Cd, Pb, Hg, Ni, V, Cr, Cu, and Zn) exists, as studies on a Mediterranean coastal system affected by gull guano revealed [64,65]. Regarding the samples from Ragusa, we suppose that this area, although it does not present industrial settlements, is affected by sea currents that convey inorganic contaminants in that direction from the nearby Augusta Bay where an important commercial, industrial and tourist port and a fundamental naval base of the Italian Navy is situated.

The evaluation of the data (Table 7) concerning the contents of potentially toxic element in fishes shows that in rays (*Raja species*), a benthic macro-invertebrate fish that could be considered as a bio-indicator of marine pollution, As levels (17.98 mg kg^−1^) are far higher than those found in other species. These results are related to the behavior of this species, to live in close connection with sediment on the sea bed and to the alimentary habit of drawing nourishment directly from marine sediments, which in this area are contaminated. However, the health impact of this result in the edible tissues of fish is difficult to interpret in terms of the ability to induce adverse effects. In fact, while the arsenic dissolved in water is mostly inorganic, in fishes, arsenic is present predominantly in the organic forms of arsenobetaine and arsenocholine, which are virtually non-toxic [66].

As mentioned before, there isn’t currently a law that cites the safety limits for As, Ni, V, Cr and Cu. Particularly, the Ni and V contents do not present significant differences among the fishes of Gela and the control; Ni was always present in higher concentrations than V, and this distribution pattern reflects the trend observed in mullets (Table 6). Therefore, we can assume that the inter-species metal capture capabilities may be a result of a similar metal accumulation. Among the other micro-constituents, for which no specific law limits currently exist, higher concentrations were observed for Cr and the data did not show significant differences among the samples from different collection areas (range between 0.26 and 0.29 mg kg^−1^). Moreover, the levels were not very different from those determined for mullets. Conversely, the distribution pattern of Cu in the present study shows a different metal accumulation: the samples from Gela had the lowest content (range: n.d–0.07 mg kg^−1^) while the highest were in sea bass (*Dicentrarchus labrax*) and steenbras (*Lithognathus mormyrus*) (0.22 and 0.21 mg kg^−1^, respectively). The different abilities of different fishes to store traces of potential toxic elements could be responsible for the differences in the Cu concentrations. However, the different Cu content could also be due to the presence of munitions—brass or bronze objects on the bottom of the sea in Patti or Ragusa—so that this element may be bio-available as a contaminant.

The comparative analysis of metal pollution among the fish species in Table 6 and Table 7 evidence that fishes from Gela showed a similar trend in potentially toxic element contamination, characterized by high Cd levels, Hg contents lower than the law limits and Pb concentrations slightly higher than the legal limits.

### 3.4. Intake Evaluation

The data determined for sea urchins, mullets, sea hens and rays has been applied to assess the health risk for the consumers. Therefore, to predict the human intake we considered the recommended dietary allowance (RDA) for Cu and Cr [67]; the maximum residue limit (MRL), the tolerable weekly intake (TWI) and the provisional tolerable weekly intake (PTWI) values, established by the Europe Food Safety Agency [68,69,70,71,72], were considered for Hg, Cd, Pb, and As. Moreover, since the EFSA CONTAM Panel determined that PTWI is no longer appropriate, in order to make these values more health protective, we determined the benchmark dose of 1% extra risk (BMDL_01_) [68,70] for As and Pb. To evaluate the chronic dietary exposure to Ni, we calculated the tolerable daily intake (TDI) based on the EFSA experts’ safe level because there are no legal maximum levels for nickel in food [73]. For vanadium, EFSA failed to establish a tolerable upper intake level because of difficulties in identifying a pivotal study to be used as a point of departure. All these parameters are reported in Table 8.

In order to evaluate the risk associated with the consumption of fish from the study area, the dietary intake was assessed for an adult (60 kg for an Italian) assuming a daily ingestion of 77.8 gr muscle meat of fish [37] and a weekly ingestion of 30 gr sea urchins; the results are reported in Table 8. As mentioned above, MRL levels for potentially toxic elements in sea urchins have not been established yet; then, we considered the maximum reside levels fixed for bivalve mollusks for Pb [44], Hg [44,57] and Cd [49].

The data analysis showed that the Hg levels were less than the MRL (0.50 mg kg^−1^) in all the species. On the contrary, with the exception of sea urchins (MRL value 1.0 mg kg^−1^), Cd concentrations in all the other samples exceeded the MRL value (0.10 mg kg^−1^). Relating to Pb, all the fish samples were just above the specific MRL (0.3 mg kg^−1^) while the sea urchins presented levels that were significantly greater than the limit in the European legislation (1.5 mg kg^−1^).

The comparative analysis of pollution among the species analyzed evidenced that all the samples do not exceed the PTWI value for Hg, although a different trend in metal contamination can be evidenced. In fact, Hg reaches the highest residual levels in mullets, rays and sea hen that live on the seabed and accumulate contaminants from sediments (particularly ray), or have carnivorous feeding habits.

Compared to the safety standards, the results of the present study show that: Cd content in sea urchins (0.45%) remain within safety margins of TWI while in all the fish samples this value was exceeded (143.41–157.21%); conversely, PTWI and the extra-safety margin of BMDL_01_ are within the safety limits for Pb in mullets (13.22% and 31.47%, respectively), sea hen (12.09% and 28.79%, respectively) and rays (11.18% and 26.62%, respectively) while BMDL_01_ in sea urchins is borderline (98.89%); Ni (1.79–15.93%) never exceeded the fixed TDI limit while the contents of Cu (n.d–0.93%) and Cr (22.86–60.49%) remain within the RDA margins. A separate comment should be made for the arsenic content. As the data show, As surpassed both PTWI and BMDL_01_ values in all the analyzed samples, reaching significantly high values for rays (PTWI = 1087.75% and BMDL_01_ range = 291.36–7769.63%). However, according to the EFSA opinion [68], data from seafood and fish, which are the major sources of arsenic in animal feed materials, did not indicate arsenic levels of concern because the toxicological risks mainly refer to inorganic arsenic, while the predominant forms in fish are the less toxic organic forms. EFSA concluded that food derived from fishes contributes only insignificantly to human exposure as the carry-over of arsenic in its inorganic form into edible tissue is low. Because the current data produced many results for total arsenic, but relatively few for inorganic arsenic, the CONTAM Panel considered the possibility of using a conversion factor which might provide an estimate of inorganic arsenic content from the total arsenic data. A problem with this sort of approach is that the relative proportion of inorganic arsenic and the ratio may vary depending on the seafood type. Therefore, because of the observed variability in the reported inorganic arsenic levels, it was not realistic to apply specific conversion factors to the total arsenic data. In our opinion, even if arsenic in fish products is mostly present as an organic compound, the contribution to the diet provided by these foods could be quite high, also because other food sources may contribute to the increase in quantities ingested up to the maximum level allowed by European regulations. The risk would be greater in the case of high consumption of fish, not uncommon in Italy, where some people, such as fishmongers, fishermen or large consumers of fishery products ingest quantities of fish, crustaceans and mollusks in much larger quantities than the average person.

### 3.5. Human Health Risks

The potential human health risks for fish consumption by the Gela population are summarized in Table 9 where the results of the target hazard quotient (THQ) and cumulative target hazard quotient (CTHQ) of potentially toxic elements are summarized with the corresponding oral reference dose (RfD_o_) established by the United States Environmental Protection Agency (USEPA).

Unfortunately, USEPA has no consensus RfD_o_ for inorganic lead and compounds, so it was not possible to calculate lead-THQ as for other chemicals. USEPA considers lead to be a special case because of the difficulty in identifying the classic “threshold” needed to develop an RfD_o_ [38]. For mercury we used the mercury chloride parameter, as indicated by USEPA (personally contacted), although fishes efficiently absorb methyl mercury, and expel it very slowly [74]. The oral RfD_o_ toxicity value for vanadium used, is derived from the oral RfD_o_ for vanadium pentoxide by factoring out the molecular weight of the oxide ion. The oral RfD_o_ toxicity value for chromium was evaluated considering that hexavalent chromium (Cr VI) is toxic and also that chromium trivalent (Cr III) exists. Therefore, the THQ was calculated for both Cr(VI) and Cr(III); moreover, according to the USEPA indications, it was assumed that the Cr(VI) to Cr(III) ratio is 1:6. The chromium THQ was also valuated for this value, knowing that this assumption may overestimate or underestimate the risk calculated. In order to determine the appropriate RfD_o_ for THQ, it was assumed that all arsenic ions were inorganic.

The estimated THQ for the detected inorganic elements did not pose unacceptable risks except for arsenic; furthermore, the contribution of inorganic lead could not be considered in the calculation, as indicated by USEPA after direct contact. As Table 9 shows, THQ values for Cd, Cr(III)_tot_, Cr(VI)_tot_, Cu, Hg, Ni and V were less than 1 for all fish specimens, indicating that there is no health risk from these elements over a life time of exposure. Among these elements the maximum target hazard quotient is for Cd in mullets (0.56146), ray (0.51867) and sea hen (0.51218), followed by Hg. For this element, the THQ values were almost comparable among the fish species (0.30688 for mullets and sea hen, 0.30256 for rays) and definitely higher than the value determined for sea urchins (0.00667). It is important to note that THQ values for As in all the specimens tested were above 1, indicating significant adverse health risk for non-carcinogenic effects. In particular, the resulting THQ value was very high for rays (77.69627), followed by mullets (38.50668) and sea hen (22.12546). These results indicate that through the ingestion of this element from all these fish species, people could experience significant health risk. As THQ deals with individual metals only, and since generally, food items contain more than one inorganic element, as detected in our samples, the cumulative risk effect was calculated as CTHQ. Like THQ, it should also not exceed 1; however, as to be expected from the THQ values, the calculated CTHQ showed unacceptable risk for habitual fish consumers. It is important to evidence that As contributed to more than 95.5 % of the non-cancer effect to the CTHQ. So, in order not to overestimate the CTHQ, we recalculated this parameter assuming that the content of inorganic arsenic was 1%. However, also in this case, as shown in Table 9, the threshold value is abundantly exceeded for as many as three fish species (ray, mullet and sea hen). Nevertheless, in relation to this hypothetical risk, as already stated above and according to EFSA’s opinion [68], the content of arsenic in fish is considered unimportant because the toxicological risks mainly refer to inorganic arsenic, while the predominant forms in fish are organic ones, arsenobetaine and arsenocholine, virtually considered non-toxic. Nevertheless, in our opinion, even if arsenic in fish products is mostly present as an organic compound, the contribution to dietary intake provided by these foods could be quite high, considering that other food sources can contribute to the increase of the quantities ingested up to the maximum level permitted by FAO/WHO [75].

## 4. Conclusions

In this study, for the evaluation of health risks, it was simply assumed that the local consumption of fish products came only from the area under investigation. The results obtained cannot be considered exhaustive as the number of samples examined is limited and in the future a broader and more extensive monitoring must be envisaged. However, this is a first screening on exposure to toxic compounds through fish consumption in the high environmental risk area of Gela, and the data obtained allow us to put forward some considerations and hypotheses.

For the analysis of health risk through the use of fishery products, three different exposure assessment procedures have been considered: evaluation according to regulations, evaluation according to a European harmonized approach using toxicological parameters and evaluation according to the USEPA approach.

First of all, it must be considered that the legislation governing the maximum levels of some contaminants in foodstuffs does not include all the analytes investigated in the present search and, therefore, the evaluation of the exposure according to regulations must be integrated with other evaluation approaches.

The findings of the study on potentially toxic elements (As, Cd, Cr, Cu, Hg, Ni, Pb and V) in fishes caught in the Gulf of Gela show a very high risk of dietary exposure in particular for cadmium, lead and arsenic. Regarding Cd and Pb (contaminants for which maximum allowed limits are defined in fish products within the European Community), all the fish samples analyzed had levels higher than the legal limits, in particular Cd. Samples of sea urchins were an exception since in these specimens, Cd had concentrations lower than the legal limits while Pb reached levels higher than the permissible limits for consumption. The high levels of Cd found in the sediments can determine a significant concentration of this element in demersal and benthic species such as the analyzed specimens, which could therefore play a role as environmental indicators.

The effect of anthropogenic activities on inorganic element loads of edible fish tissues reveals alarming arsenic content. The results from this study showed that the contamination of sediments by arsenic exceeded the threshold limit stated by the European Regulations. Therefore, we believe that under such conditions, the accumulation of this potentially toxic element in fishes could represent an unacceptable risk for the potential long-term impact on public health and ecosystem integrity. With the evaluation in accordance to the European harmonized approach, the exceeding of the limits set for cadmium in some monitored matrices was observed while a hypothetical risk for arsenic was highlighted.

From the human health point of view, the THQ and CTHQ values indicate significant adverse health risks of non-carcinogenic effect. Moreover, these data were unfortunately calculated, according to the USEPA approach, without considering the lead contribution that often results above the legal limit.

The results of the research provide an initial confirmation of the epidemiological hypotheses that also indicate one of the most important factors of exposure to potentially toxic elements in the consumption of local fish products. More generally, the levels of dietary exposure are even more serious if we consider that, in addition to the individual fishes analyzed, it is necessary to add the contribution of potentially toxic elements coming from the other foods that constitute a diet (water, meat, dairy products, etc.).

## Figures and Tables

**Table 1:** Sampling and physical characteristics of marine sediments.

Sample	GPS Coordinates		Color	Odor	Sediment Type
SED 1	37°2′54.78″ N	14°16′1.81″ E	brown	odorless	silty-sandy
SED 2	37°3′6.89″ N	14°15′53.54″ E	gray-black	hydrogen sulfide/hydrocarbons	silty-sandy

**Table 2:** Analytical performance of the method.

Element	R^2^	LOD ^1^ (µg L^−1^)	LOQ ^2^ (µg L^−1^)
Cr	0.99999	0.006	0.02
Ni	0.99998	0.002	0.007
As	0.99997	0.0004	0.001
Cd	0.99999	0.0005	0.002
V	0.99995	0.0009	0.003
Hg	0.99997	0.0009	0.003
Pb	0.99993	0.0004	0.001
Cu	0.99998	0.004	0.01

^1^ LOD, limit of detection (3.3 σ/S); ^2^ LOQ, limit of quantification (10 σ/S).

**Table 3:** Accuracy and precision performance for the certified matrices.

	Certified Matrix IAEA-407		Certified Matrix GWB 07402		Certified Matrix IAEA-407		Certified Matrix GWB 07402	
Element	Recovery(%)	Accuracy(RSD%)	Recovery(%)	Accuracy(RSD%)	Restricted Repeatability (RSD%)	Intermediate Repeatability (RSD%)	Restricted Repeatability (RSD%)	Intermediate Repeatability (RSD%)
Cr	94	4.22	96	3.89	1.3	5.4	3.6	7.0
Ni	97	4.96	94	5.62	0.8	2.7	2.3	3.0
As	89	3.98	88	3.56	1.5	6.9	3.3	4.3
Cd	101	5.41	112	2.96	3.7	7.1	2.3	8.6
V	88	3.98	97	4.26	3.1	4.8	3.6	7.5
Hg	92	3.06	87	2.54	1.1	5.5	3.8	9.5
Pb	75	3.42	90	1.45	2.9	3.9	3.8	8.3
Cu	92	7.23	97	5.42	3.6	4.8	2.9	8.7

**Table 4:** Concentration of potentially toxic inorganic contaminants in sediments (mg kg^−1^).

Element	SED 1	SED 2	EQS-AA ^1^
As	10.76 ± 0.31	13.44 ± 0.35	12
Ni	5.37 ± 0.04	11.11 ± 0.07	30
Hg	0.02 ± 0.00	0.01 ± 0.00	0.3
V	14.89 ± 0.16	27.17 ± 0.20	-
Pb	2.77 ± 0.01	4.53 ± 0.01	30
Cd	0.55 ± 0.04	0.60 ± 0.02	0.3
Cu	2.77 ± 0.06	4.33 ± 0.10	-
Cr	6.50 ± 0.01	14.25 ± 0.02	50

^1^ Environmental Quality Standards—Annual Average. Legislative Decree 8 November 2010 n. 260. Table 2/A, and Table 3/B.

**Table 5:** Mean values and standard deviation of concentration (mg kg^−1^ wet weight) of inorganic contaminants in 30 sea urchins samples collected from 3 different areas in Sicily.

Element	Gela	Marina Di Ragusa	Piraino	Limit Value ^1^
As	6.17 ± 0.95	4.11 ± 0.08	2.71 ± 0.07	-
Ni	0.75 ± 0.07	1.18 ± 0.10	n.d. ^2^	-
Hg	0.03 ± 0.01	n.d.	n.d.	0.5
V	n.d.n.d.	0.94 ± 0.09	n.d.	-
Pb	22.25 ± 2.40	1.59 ± 0.16	n.d.	1.5
Cd	0.02 ± 0.01	1.22 ± 0.10	n.d.	1.0
Cu	2.31 ± 0.15	0.27 ± 0.04	0.83 ± 0.08	-
Cr	2.29 ± 0.52	0.93 ± 0.07	n.d.	-

^1^ Limit values established by the Commission Regulation (EC) n.1881/2006 for Pb and Hg levels in bivalve mollusks. Limit values established by the Commission Regulation (EU) No 488/2014 for Cd levels in bivalve mollusks. ^2^ n.d.: lower than the limit of detection (LOD).

**Table 6:** Concentration (mg kg^−1^ wet weight, mean ± SD) of potentially toxic elements in mullets (*Mullus barbatus*) samples from Gela and compared with samples from different Mediterranean areas.

Sample	Sites	Weight	Mean Concentration ± SD							
		(gr)	As	Ni	Hg	V	Pb	Cd	Cu	Cr
Mullet 1	Gela	60	8.40 ± 0.07	0.27 ± 0.04	0.07 ± 0.01	0.14 ± 0.01	0.31 ± 0.05	0.37 ± 0.05	0.03 ± 0.00	0.24 ± 0.03
Mullet 2	Gela	90	9.42 ± 0.29	0.41 ± 0.05	0.07 ± 0.00	0.20 ± 0.01	0.41 ± 0.01	0.50 ± 0.01	0.04 ± 0.01	0.38 ± 0.02
Minimum value	Gela	90–60	8.10 ± 0.06	0.22 ± 0.04	0.07 ± 0.01	0.12 ± 0.01	0.29 ± 0.02	0.35 ± 0.03	0.03 ± 0.00	0.21 ± 0.01
Maximum value	Gela	90–60	9.68 ± 0.12	0.45 ± 0.05	0.07 ± 0.00	0.23 ± 0.03	0.43 ± 0.04	0.56 ± 0.06	0.04 ± 0.01	0.40 ± 0.04
Mean value	Gela	90–60	8.91 ± 0.72	0.34 ± 0.10	0.07 ± 0.00	0.17 ± 0.03	0.36 ± 0.07	0.43 ± 0.09	0.04 ± 0.01	0.31 ± 0.10
Limit vaue ^1^					1.0		0.30	0.050		
Storelli and Marcotigiano (2005) [53]	Roccella Ionica	50	-	-	0.12 ± 0.10	-	0.13 ± 0.07	0.17–0.80	-	0.29 ± 0.21
Storelli and Marcotigiano (2005) [53]	Catania	50	-	-	0.11 ± 0.10	-	0.18 ± 0.09	0.20–0.25	-	0.21 ± 0.02
Storelli and Marcotigiano (2005) [53]	Capo Passero	50	-	-	0.81 ± 0.52	-	n.d.^2^	0.22–0.45	-	0.31 ± 0.11
Kljakovic et al. (2002) [55]	Croatia	10–193	-	-	-	-	0.057–0.158	-	-	
Ferrara and Furnari (2004) [54]	Adriatic Sea	20–45	-	-	-	-	0.0088–0.029	-	-	0.006–0.026
Naccari et al. (2015) [56]	Mediterranean FAO zone 37 1.3	40–100	-	-	<0.06	-	<0.09	<0.02	-	-

^1^ Limit values established by the Commission Regulation (EC) n.1881/2006 expressed in mg kg^−1^ of wet weight. Maximum levels reported regards muscle meat of fish and rays (*Raja species*). The regulation concerns the Pb and Hg levels. Limit values established by the Commission Regulation (EC) No 629/2008 expressed in mg kg^−1^ of wet weight. Maximum levels reported regard muscle meat of fish. The regulation concerns the Hg levels in some fish species. The values established in this regulation coincide with those of the previous regulation. Limit values established by the Commission Regulation (EU) No 488/2014 expressed in mg kg^−1^ of wet weight. Maximum levels reported regard muscle meat of fish. The regulation concerns the Cd levels. ^2^ n.d.: lower than the limit of detection (LOD).

**Table 7:** Concentration (mg kg^−1^ wet weight, mean ± SD) of potentially toxic elements in fish samples from Gela Gulf compared to samples from two different Sicilian areas.

Samples	Sites	Length	Mean Concentration ± SD							
		(cm)	As	Ni	Hg	V	Pb	Cd	Cu	Cr
Sea hen	Gela	25–30	5.12 ± 0.09	0.33 ± 0.01	0.07 ± 0.00	0.15 ± 0.00	0.33 ± 0.01	0.39 ± 0.01	n.d.^1^	0.29 ± 0.04
Ray	Gela	36	17.98 ± 0.43	0.30 ± 0.01	0.07 ± 0.00	0.14 ± 0.01	0.31 ± 0.01	0.40 ± 0.01	0.07 ± 0.01	0.26 ± 0.02
Steen bras	Ragusa	22–25	5.06 ± 0.15	0.30 ± 0.02	0.08 ± 0.01	0.19 ± 0.01	0.31 ± 0.01	0.37 ± 0.01	0.21 ± 0.02	0.29 ± 0.02
Sea bass	Patti	20–28	0.78 ± 0.02	0.32 ± 0.02	0.03 ± 0.01	0.19 ± 0.01	0.30 ± 0.01	0.37 ± 0.01	0.22 ± 0.01	0.29 ± 0.01
Sea bream	Patti	18–22	1.10 ± 0.10	0.29 ± 0.01	0.02 ± 0.00	0.14 ± 0.01	0.29 ± 0.01	0.37 ± 0.04	0.10 ± 0.01	0.26 ± 0.02
Limit value ^2^			-	-	1.0	-	0.30	0.050	-	-

^1^ n.d.: lower than the limit of detection (LOD). ^2^Limit values established by the Commission Regulation (EC) n.1881/2006 expressed in mg kg^−1^ of wet weight. Maximum levels reported regards muscle meat of fish and rays (*Raja species*). The regulation concerns the Pb and Hg levels. Limit values established by the Commission Regulation (EC) No 629/2008 expressed in mg kg^−1^ of wet weight. Maximum levels reported regard muscle meat of fish. The regulation concerns the Hg levels in some fish species. The values established in this regulation coincide with those of the previous regulation. ^2^ Limit values established by the Commission Regulation (EU) No 488/2014 expressed in mg kg^−1^ of wet weight. Maximum levels reported regards muscle meat of fish. The regulation concerns the Cd levels.

**Table 8:** Estimated metals intake of daily seafood consumption (4 gr of sea urchin and 77.8 gr of fish muscle) for an adult of 60 kg.

Element	As	Ni	Hg	Pb	Cd	Cu	Cr
MRLfor bivalve mollusks (mg/Kg/day)			0.50	1.5	1.0		
MRLfor muscle meat of fish (mg/Kg/day)			0.50	0.30	0.10		
TWI or PTWI(mg/kg b.w./week)	0.015		0.004	0.025	0.0025		
TDI (µg/kg b.w./day)		2.8					
BMDL_01_(µg/kg b.w./day)	0.3–8			1.5			
RDA (mg/day)						1.00	0.04
**Sea Urchin** (mean value mg/Kg)	6.17	0.75	0.03	22.25	0.02	2.31	2.29
% of TWI or PTWI estimated by mean value	19.2		0.35	41.53	0.45		
% of TDI estimated by mean value		1.79					
% of BMDL_01_estimated by mean value	5.14–137.11			98.89			
% of RDA estimated by mean value						0.93	22.86
**Mullet** (mean value mg/Kg)	8.91	0.34	0.07	0.36	0.43	0.04	0.31
% of TWI or PTWI estimated by mean value	539.09		16.11	13.22	157.21		
% of TDI estimated by mean value		15.93					
% of BMDL_01_estimated by mean value	144.40–3850.67			31.47			
% of RDA estimated by mean value						0.29	60.49
**Ray** (mean value mg/Kg)	17.98	0.30	0.07	0.31	0.40	0.07	0.26
% of TWI or PTWI estimated by mean value	1087.75		16.11	11.18	145.23		
% of TDI estimated by mean value		13.89					
% of BMDL_01_ estimated by mean value	291.36–7769.63			26.62			
% of RDA estimated by mean value						0.58	51.15
**Sea hen** (mean value mg/Kg)	5.12	0.33	0.07	0.33	0.39	n.d. ^1^	0.29
% of TWI or PTWI estimated by mean value	309.76		16.11	12.09	143.41		
% of TDI estimated by mean value		15.28					
% of BMDL_01_ estimated by mean value	82.97–2212.55			28.79			
% of RDA estimated by mean value						n.d.	56.99

n.d.: lower than the limit of detection (LOD); b.w.= body weight.

**Table 9:** Estimated target hazard quotient (THQ) and cumulative target hazard quotient (CTHQ) of inorganic elements for the inhabitants of the study area through the consumption of fishes.

	RfDo ^1^	Sea Urchin	Mullet	Ray	Sea Hen
	(mg/kg-day)				
As	0.00030	137.111	3850.668	7769.627	2212.546
Ni	0.02000	0.00251	0.02230	0.01945	0.02140
Hg	0.00030	0.00667	0.30688	0.30256	0.30688
V	0.00504	0.00000	0.04348	0.03679	0.03833
Cd	0.00100	0.00160	0.56146	0.51867	0.51218
Cu	0.04000	0.00386	0.00120	0.00243	0.00000
Cr (III)	150.000	0.00010	0.00027	0.00023	0.00025
Cr (VI)	0.00300	0.05080	0.13442	0.11367	0.12664
Cr (VI) ^2^	0.00300	0.00847	0.02248	0.01902	0.02118
CTHQ ^3^		139.421	3946.447	7859.518	2302.542
CTHQ ^4^		0.03681	134.286	167.587	112.122

^1^ Oral reference dose (RfDo). ^2^ This value was calculated according to the USEPA indication and assuming that the Cr(VI) to Cr(III) ratio is 1:6 (USEPA. 2018). ^3^ This parameter was calculated considering for chromium the value reported for Cr(VI). ^4^ This parameter was calculated assuming an inorganic arsenic content equal to 1% of the total value.
